# Association between vitamin D level and bronchopulmonary dysplasia: A systematic review and meta-analysis

**DOI:** 10.1371/journal.pone.0235332

**Published:** 2020-07-06

**Authors:** Hye Won Park, Gina Lim, Yong-Mean Park, Misoo Chang, Jae Sung Son, Ran Lee

**Affiliations:** 1 Department of Pediatrics, Konkuk University Medical Center, Seoul, Republic of Korea; 2 Konkuk University School of Medicine, Seoul, Republic of Korea; 3 Department of Pediatrics, Ulsan University Hospital, University of Ulsan College of Medicine, Ulsan, Republic of Korea; 4 Research Coordinating Center, Konkuk University Medical Center, Seoul, Republic of Korea; Center of Pediatrics, GERMANY

## Abstract

Neonatal vitamin D deficiency is common and is associated with development of pulmonary disease in children and adults. While the role of vitamin D in normal lung development is well established, the association between vitamin D deficiency and bronchopulmonary dysplasia (BPD) remains unclear. The present meta-analysis was conducted to evaluate the relationship between vitamin D and BPD. We identified relevant studies (n = 8) using the PubMed, EMBASE, Cochrane Library, and KoreaMed databases and applied the Newcastle–Ottawa Scale to assess the methodological components of each study, and used I^2^ statistic to evaluate heterogeneity. Comprehensive Meta-Analysis software version 3.3 was used for the statistical analysis. A total of 909 infants were included, of whom 251 (27.6%) were diagnosed with BPD. We found that both vitamin D deficiency at birth (four studies; OR 2.405; 95% CI 1.269 to 4.560; p = 0.007) and low levels of vitamin D at birth (four studies; standardized mean difference -1.463; 95% CI -2.900 to -0.027; p = 0.046) were associated with BPD. The compiled data suggest that antenatal vitamin D deficiency and low vitamin D levels are associated with neonatal BPD.

## Introduction

Vitamin D is a fat soluble vitamin whose active form, 1,25-dihydroxyvitamin D (1,25[OH]2D), is essential for calcium and phosphorus absorption as well as bone mineralization [1, 2]. Vitamin D receptors are expressed by most cell types [1, 3] and recent evidence also supports roles for vitamin D in cardiovascular disease, chronic respiratory disease, infection, autoimmune disease, and low birth weight, or preterm birth [1, 3–7].

Bronchopulmonary dysplasia (BPD) was first defined by Northway in 1967 in preterm infants with respiratory distress syndrome following prolonged ventilator support [8]. In its classic form, BPD in preterm infants is characterized by airway injury and parenchymal fibrosis leading to chronic respiratory failure and a prolonged oxygen requirement, similar to chronic obstructive pulmonary disease in adults [9, 10]. BPD in extremely low birth weight infants following the use of surfactant and antenatal steroid was characterized by Jobe [10, 11] as arrest of lung development in both alveolar and vascular development. This disruption of the developmental process occurs during or prior to the late canalicular and saccular stages [9, 12].

Vitamin D deficiency is common in preterm and full term infants [13–16] and is associated with pulmonary diseases such as asthma or respiratory infection in children [17, 18] and adults [19]. Vitamin D deficiency affects lung alveolar and vascular development, immune modulation, repair, and function [4, 5, 19–32]. Recently, the role of vitamin D in normal lung development and in BPD was characterized [20, 33, 34], but the association between vitamin D deficiency and BPD remains controversial.

Thus, we conducted a meta-analysis to assess the relevance of vitamin D deficiency or vitamin D level at birth or within the 24 hours after birth that reflect the serum vitamin D levels of the fetus and mothers to BPD.

## Methods

### Search strategy and study selection

We searched PubMed, EMBASE, Cochrane Library, and KoreaMed databases using the search terms: “vitamin D” or “25-hydroxyvitamin D” or “25-hydroxyergocalciferol” or “ergocalciferol” or “cholecalciferol” or “hydroxycholecalciferol” or “calcifediol” or “dihydroxycholecalciferol” or “25(OH)D” or “1,25(OH)2-vitD”; and “bronchopulmonary dysplasia” or “chronic lung disease” or “lung injury”; and “prematurity” or “low birth weight infant” or “neonate”. The detailed search strategy for PubMed is presented in **S1 Table.** There were no restrictions on language, population, or publication year. The last search was performed on June 24, 2019. We initially screened the study titles and abstracts, and subsequently reviewed the full-text articles. Articles were independently reviewed by two reviewers (authors HW Park and G Lim), who applied selection criteria to determine article eligibility for inclusion in the meta-analysis.

### Inclusion and exclusion criteria

We included randomized controlled trials, observational studies (including case-control studies), cohort studies, and cross sectional studies in our analysis. BPD was defined as an oxygen dependency at either 28 days of age or 36 weeks of postmenstrual age. BPD was diagnosed in each study according to either the National Institutes of Health consensus [35–38], or other criteria for oxygen dependency, at 36 weeks of postmenstrual age [39], or at 28 days of age [40, 41]. Case reports, case series, single-arm cohort studies, and animal studies were excluded from the meta-analysis.

### Data extraction and study quality assessment

Data were independently extracted from the full-text versions of selected studies by the authors (HW Park and G Lim). The collected data included first author name, publication year, study design, study location, study period, study population, time of vitamin D level measurement, definition of BPD, sample size, BPD incidence, and vitamin D level. We also assessed the quality of the included studies using the Newcastle–Ottawa Scale [42]. The Newcastle–Ottawa Scale is based on a “star system” and is composed of eight items evaluating three domains: selection (four items), comparability (one item), and outcomes (three items). One star is awarded for each item, with the exception of the comparability item, which may receive two stars. Using this scale, articles were assigned a quality score between 0 and 9. Based on their total scores, studies were categorized as ‘low quality’ (≤ 3), ‘moderate quality’ (4–5), and high quality (≥ 6). Any disagreements regarding data interpretation or quality assessment were resolved by discussion with a third reviewer (R Lee); any such study was subsequently reevaluated.

### Data synthesis and statistical analyses

The results are presented as summary odds ratios (ORs) with 95% confidence intervals (CIs) for dichotomous data, and as the standardized mean difference (SMD) and 95% CIs for continuous variables, to demonstrate the relationship between vitamin D and BPD. Among four studies that reported the relationship between vitamin D levels and BPD, three reported the means and standard deviations (SDs) of the 25(OH)D level (a continuous variable); one study did not [43], reporting instead the median and interquartile range (IQR). We tried to contact the author to get the data expressed as mean ± SD to allow the data to be included.

Forest plots were generated to assess between-study heterogeneity. The I^2^ statistic was used to determine the percentage of variation across studies. The I^2^ statistic indicates whether the heterogeneity among studies is low (25–50%), moderate (50–75%) or high (>75%). Regardless of the degree of heterogeneity, a random effects model was used for analysis, which is more conservative and has wider CIs than a fixed effect model [44, 45]. Inverse variance weighting was used for weighting the random effects model in this study.

A sensitivity analysis was performed by removing the results of individual studies from the data set and subsequently evaluating the robustness of the combined estimates and the contribution of each study to the pooled OR. To detect temporal trends, a cumulative analysis was performed by adding studies one at a time according to the date of publication.

We performed the Begg and Mazumdar rank correlation test and Egger’s regression test to evaluate publication bias. Publication bias was also evaluated using a funnel plot, which shows the distribution of the effect sizes against the standard error values. The meta-analysis was performed using Comprehensive Meta-Analysis software (version 3.3; Biostat, Englewood, NJ, USA).

## Results

### Literature search and study selection

**Fig 1:**
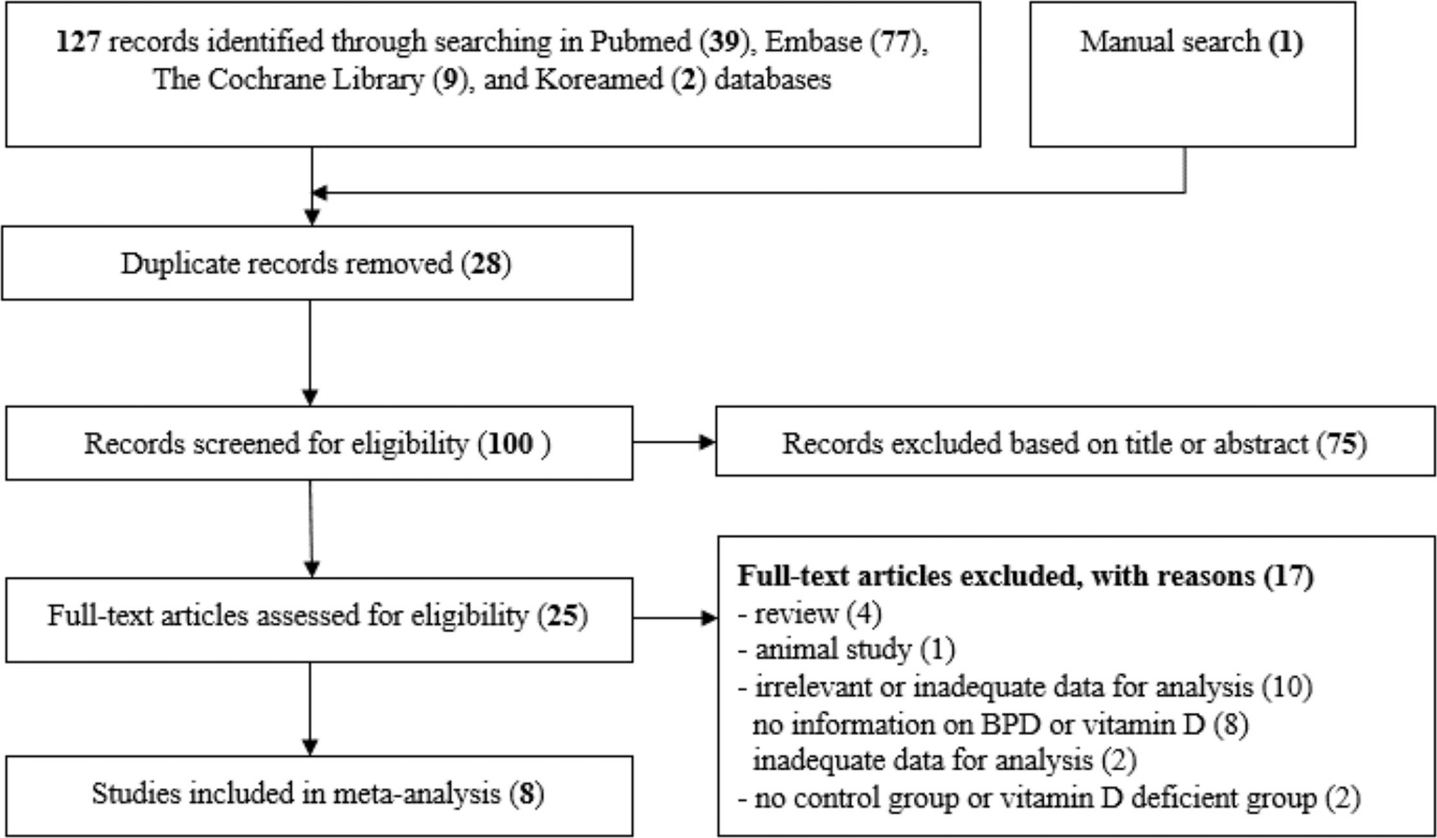
Flow diagram for study selection.

The study selection process and exclusion criteria are described in Fig 1. Of the 128 studies identified initially, 120 were excluded based on review of the title, abstract or full text. The reasons for excluding 17 studies [33, 34, 46–60] after full-text review, are provided in Fig 1. The remaining eight studies met the inclusion criteria and were included in the meta-analysis [35, 37, 38, 40, 41, 43, 61, 62].

### Characteristics of the included studies

A total of 909 infants were included in this meta-analysis. The mean birth weight of the infants was 1,322.4g and the mean gestational age of all infants (with and without BPD) was 29.1 weeks. One study did not include data regarding gestational age at birth [61]. The characteristics of the study populations are described in Table 1. Among 909 infants, 251 (27.6%) infants were diagnosed with BPD (Table 1). Two studies [40, 41] defined BPD as an oxygen dependency at 28 days of age; the remaining studies [35, 37, 38, 43, 61, 62] defined BPD as oxygen dependency at 36 weeks of postmenstrual age, based on National Institutes of Health consensus [63]. The incidence of BPD vary from 6.6% to 56.8%, 6.6% in the study of Yang et al [61], and 56.8% in the study of Kazzi et al. [38].

**Table 1:** Characteristics of studies included in this meta-analysis.

Studies	Study design	Population	Time of vitamin D measurement	Definition of BPD (oxygen dependency)	Definition of vitamin D deficiency (25 (OH) D level)	Study population GA (week), BW (g) expressed as mean ± SD or mean	NOS
Onwuneme, 2015	Prospective	GA < 32 weeks or birth weight < 1,500 g	Within 24 h of birth	at 36 weeks of PMA	< 12 ng/mL (30 nmol/L)	**All (n = 94),** GA: 28.8 ± 2.09, BW: 1193 ± 375	8
						**BPD (n = 34**)	
						**Vitamin D <30nmol/L (n = 60)** GA: 28.6 ± 2.3, BW: 1171 ± 363	
						**Vitamin D ≥30nmol/L (n = 34)** GA: 29.1 ± 1.71, BW: 1229 ± 398	
Yu, 2017	Prospective	GA < 34 weeks	On admission	at 28 days of age	< 20 ng/mL	**All (n = 260), BPD (n = 41)**	8
						**Non-BPD group (n = 219)** GA: 31.5 ± 1.6, BW: 1734 ± 359	
						**BPD group (n = 41)** GA: 28.0 ±1.6, BW: 1141 ± 242	
Kazzi, 2018	Prospective	Birth weight ≤ 1,250 g	Within 24 h of birth	at 36 weeks of PMA	≤ 10 ng/mL	All (n = 89; 81^*^), BPD (n = 46)	8
						**Vitamin D ≤10 ng/mL group (n = 32)** GA: 27 ± 2, BW: 860 ± 262	
						**Vitamin D >10 ng/mL group (n = 57)** GA: 27 ± 2, BW: 873 ± 210	
Kim, 2019	Retrospective	Birth weight < 1,500 g	Within 24 h of birth	at 28 days of age	< 20 ng/mL	**All (n = 188),** GA: 28.4 ± 3.0, BW: 1104 ± 298.1	8
						**BPD (n = 55)**	
						**Vitamin D <10 ng/mL (n = 83)** GA: 28.3 ±3.3, BW: 1045.2 ± 293.8	
						**Vitamin D 10–20 ng/mL (n = 67)** GA: 28.5 ± 3.2, BW: 1098.3 ± 297.4	
						**Vitamin D ≥20 ng/mL (n = 38)** GA: 29.1 ± 2.5, BW: 1245.7 ± 267.1	
Cetinkaya, 2015	Prospective	GA ≤ 32 weeks	On admission	at 36 weeks of PMA	Measured value**†**	**All (n = 100),** GA: 28.4, BW: 1006.5	6
						**Non-BPD group (n = 69)** GA: 28.9 ±2.46, BW: 1063.8 ± 251.1	
						**BPD group (n = 31)** GA: 27.2 ± 2.4, BW: 875.6 ± 247.3	
Joung, 2016	Prospective	GA< 29 weeks	At birth	at 36 weeks of PMA	Measured value**†**	**All (n = 44),** GA: 26.6, BW: 870	7
						**BPD group (n = 18)**	
Mao, 2018	Prospective	GA ≤ 32 weeks	Within 24 h of birth	at 36 weeks of PMA	Measured value**†**	**All (n = 39),** GA: 29.5, BW: 1268.9	9
						**Non-BPD group (n = 20)** GA: 29.8 ± 0.2, BW: 1323 ± 51.9	
						**BPD group (n = 19)** GA: 29.3 ± 0.3, BW: 1212 ± 50.5	
Yang, 2018	Prospective	GA < 37 weeks	Within 24 h of birth	at 36 weeks of PMA	Measured value**†**	**All (n = 106), BPD (n = 7)**	5
						NA/ 1877.5	

* BPD outcome was checked in 81 infants.

† the measured value of vitamin D was used in the analysis

**Abbreviations:** GA, gestational age at birth; BW, birth weight; BPD, bronchopulmonary dysplasia; NOS, Newcastle–Ottawa Scale; PMA, postmenstrual age

Among eight studies, four [38, 40, 41, 62] presented results on the association of vitamin D deficiency with BPD; the other four studies [35, 37, 43, 61] assessed the association of vitamin D level with BPD. The four studies that evaluated vitamin D deficiency used 25(OH)D cutoff values of 20 ng/mL(50 nmol/L) [40, 41], 10 ng/mL [38], or 12 ng/mL(30 nmol/L) [62]. Vitamin D levels were measured from cord blood or at the time of hospital admission or in the 24 hours before intravenous or oral vitamin D administration.

The results of quality assessment of included studies according to the Newcastle–Ottawa Scale are shown in **Table 1**.

### Pooled meta-analysis results

**Fig 2:**
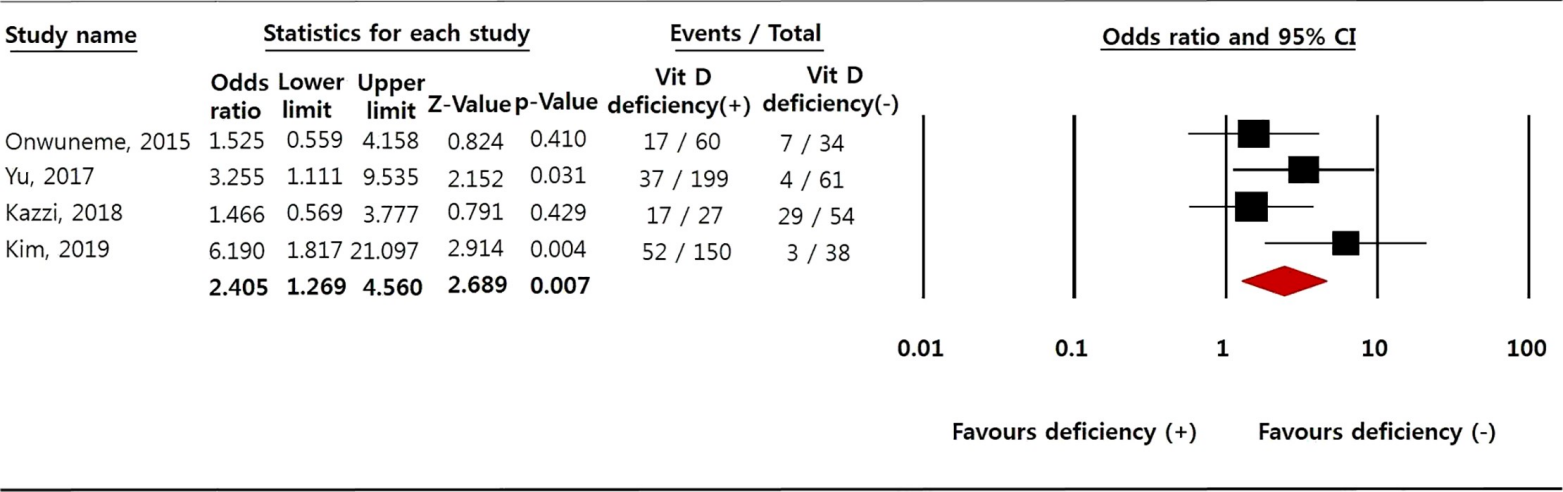
Meta-analysis of the relationship between vitamin D deficiency and bronchopulmonary dysplasia. Forest plot of the random effects model, diamonds indicate the effect size of given study, which is proportional to the weight of the study.

A significant association was detected between vitamin D deficiency and BPD based on oxygen dependency at 28 days of age or at 36 weeks of corrected age (OR 2.405; 95% CI 1.269 to 4.560; p = 0.007; **Fig 2**), and there was no significant heterogeneity among the studies (p = 0.22; I^2^ = 32.1%). However, as only four studies were included in this part of the meta-analysis, we used a random effects model [44, 45]. Publication bias was not detected by the Begg and Mazumdar rank correlation test (p = 0.089). However, the Egger’s regression test (p = 0.030) and asymmetric funnel plot **(S1 Fig)** indicated possible publication bias. Thus, we performed a trim and fill adjustment, which yielded the same results (OR 2.405; 95% CI 1.269 to 4.560). The sensitivity analysis showed that no single study changed the pooled results (**S2 Fig**) and the results of cumulative analysis indicated no temporal effects (**S2 Fig**).

**Fig 3:**
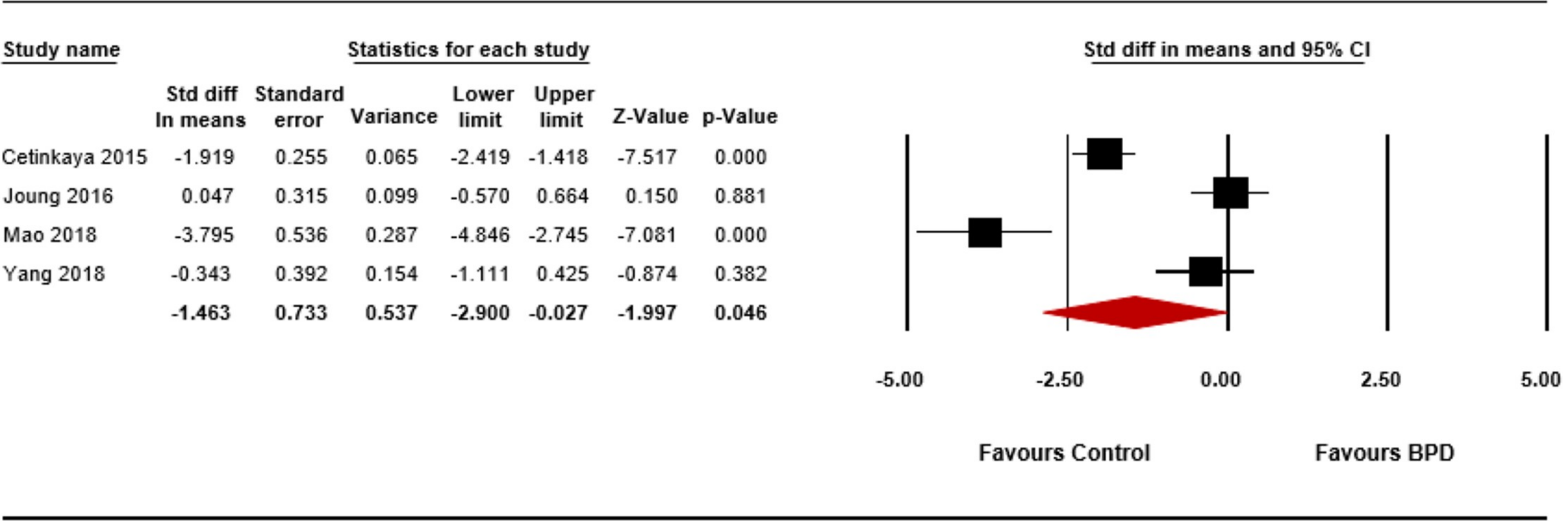
Meta-analysis of the relationship between vitamin D level and bronchopulmonary dysplasia. Forest plot of the random effects model, diamonds indicate the effect size of given study, which is proportional to the weight of that study. Std; standardized, BPD; bronchopulmonary dysplasia.

Vitamin D levels were significantly lower in the BPD infants compared with the controls (SMD = -1.463; 95% CI -2.900 to -0.027; p = 0.046; **Fig 3**). The heterogeneity assessment indicated good heterogeneity among the studies (p < 0.001; I^2^ = 94.20%). Due to the small sample size, publication bias could not be assessed by the funnel plot (**S3 Fig**). The Begg and Mazumdar rank correlation test (p = 0.734) and Egger’s regression test (p = 0.756) indicated no publication bias. The sensitivity analysis (**S4 Fig**) and cumulative analysis (**S4 Fig**) showed that the exclusion of no single study significantly changed the pooled results.

## Discussion

The half-life of 25(OH)D is longer than that of 1,25(OH)_2_D (15 days vs 10–20 hours [2]) and 1,25(OH)_2_D serum levels are influenced by factors other than vitamin D status including, parathyroid hormone, calcium and phosphorus level [46]. Levels of 25(OH)D, which is the most frequently used indicator of vitamin D status [1], were measured at birth or within the 24 hours after birth in all included studies. Serum levels of 25(OH)D measured in the 24 hours before vitamin D supplementation correlated with the serum vitamin D levels of the fetus [38, 41] and the mother [15, 16, 38, 41, 64, 65], which is likely attributable to maternal transfer of vitamin D to the fetus [41].

Vitamin D deficiency has been reported in 40–50% of pregnant females and 45–60% of preterm infants [66–69]. In our meta-analysis, vitamin D deficiency was observed in 70% (range 33–80%) of preterm infants. Holick et al. [1] defined vitamin D deficiency as a serum 25(OH)D level of less than 20 ng/mL(50 nmol/L). Our analysis included two studies [40, 41] that used 20 ng/mL as the cutoff, one study [38] that used 10 ng/mL (based on the National Institute of Health Office of Dietary Supplements recommendations [46]), and one study [62] that used 12 ng/mL (30 nmol/L) (based on the Institute of Medicine report [70]) (**Table 1**). Four studies did not provide definitions of deficiency and, instead, reported levels of vitamin D [35, 37, 43, 61].

The etiology of BPD is multifactorial [63, 71], thus vitamin D deficiency may not be the only cause of BPD. However, the important role of nutrition in prenatal and postnatal lung growth has been demonstrated in previous reports [57, 59, 72]. Vitamin D has roles in lung development in anatomical [20–22, 25, 26], functional [21, 22, 27, 28, 73], and immunological terms [5, 21, 29], as well as in the development of BPD [20, 74–76] and ther recovery therefrom [55].

In animal studies, vitamin D deficiency during lung development was associated with inhibition of alveolar type II cell and fibroblast proliferation, reduced surfactant or antioxidant production, and upregulation of vitamin D receptors [20, 25, 26]. Antenatal vitamin D deficiency is linked to impaired anatomical lung development, including reduced tracheal diameter, irregular cartilage [21, 22], increased airway smooth muscle mass and elevated collagen synthesis [20, 21, 25], as well as to altered pulmonary function, including increased hyperresponsiveness, increased resistance and decreased compliance, obstructive lung disease, and low performance on pulmonary function tests, such as forced vital capacity or forced expiratory volume [21, 22, 27, 28, 73]. Airway inflammation is also found in case of antenatal vitamin D deficiency, demonstrated by elevated neutrophil and decreased lymphocyte counts in bronchoalveolar lavage [21], as well as by decreased expression of IkBa through NFKBIA, and by other indicators of postnatal airway inflammation [5, 21, 29].

The vitamin D pathway also contributes to impaired lung development after endotoxin exposure, where vitamin D receptors and vitamin D catabolic enzymes promote a deficiency-like state [20]. Vitamin D deficiency could contribute to BPD development after endotoxin exposure through increased expression of CYP24A1, a vitamin D regulatory enzyme, and decreased expression of vitamin D receptors and 1α-OHase in the lung [20], and of VEGF expression and secretion, thereby impairing the processes of angiogenesis and vasculogenesis [74–76]. Vitamin D supplementation may help to restore proper alveolar development through suppression of interferon-γ [55].

There have also been studies [25, 73, 77, 78] including systematic reviews reporting a role of vitamin D in fetal and neonatal lung maturation [25, 73, 77] and fetal outcomes during pregnancy [78]. However, these studies did not report the relationship between vitamin D and BPD, but rather the association of vitamin D levels with respiratory infections, and asthma in offspring [78], low birth weight [25, 78], or preterm birth, duration of ventilator support, and duration of oxygen supplementation [25]. In this meta-analysis, we found a significant association between BPD and vitamin D deficiency (OR 2.405; 95% CI 1.269 to 4.560; p = 0.007; **Fig 2**), and between BPD and low 25(OH)D levels at birth (SMD = -1.463; 95% CI -2.900 to -0.027; p = 0.046); **Fig 3**). Several studies also reported relationships of vitamin D with BPD and lung development.

There were some limitations to this study. First, as this meta-analysis included a small number of trials (only four studies of vitamin D deficiency and four of vitamin D levels), we must cautiously interpret the results regarding publication bias of the Begg and Mazumdar rank correlation test, Egger’s regression test, and funnel plot (S1 Fig and S3 Fig). Therefore, we did sensitivity analysis and cumulative analysis according to date of publication, and the results showed no temporal effects. Second, although we did not detect significant heterogeneity among the studies (p = 0.22; I^2^ = 32.1%) in the analysis of the relationship between vitamin D deficiency and BPD, the possibility of heterogeneity remains. However, we performed the analysis using a random-effect model, which is more conservative, to generate a more accurate estimate with wider CIs [44, 45], compare with fixed effect model.

Our meta-analysis indicated that the vitamin D level at birth, which reflects fetal and maternal vitamin D status during pregnancy, was significantly associated with BPD incidence. Antenatal vitamin D promotes critical lung development during the canalicular and saccular stages and maternal vitamin D supplementation during pregnancy and for preterm infants is essential for ensuring optimal levels in the fetus, reducing the risk of BPD by promoting healthy lung development. Although postnatal vitamin D supplementation is unable to completely reverse the lung and airway defects caused by fetal vitamin D deficiency, we believe that it may promote postnatal lung development, at least during the first 2 years of life, during which alveolar development occurs [22, 79, 80], as well as reduce airway hyperresponsiveness and inflammation [5, 21, 29]. The dose of vitamin D used as a supplement in preterm and very low birth weight infants varies; 200–400 IU/d is recommended by the American Academy of Pediatrics [81] and 800–1,000 IU/d by the European Society of Pediatric Gastroenterology [82]. A recent meta-analysis [83] found that neither 25(OH)D levels nor BPD incidence differed between these dose ranges. Additional studies are needed to fully evaluate the effects of vitamin D supplementation on maternal and offspring health, including prevention of BPD.

## Supporting information

S1 Table MEDLINE search strategy. (DOCX)

S1 Fig Funnel plot for the relationship between vitamin D deficiency and bronchopulmonary dysplasia. An asymmetrical funnel plot is displayed.
(TIF)

S2 Fig Sensitivity analysis (2–1) and cumulative analysis (2–2) for the relationship between vitamin D deficiency and bronchopulmonary dysplasia. (TIF)

S3 Fig Funnel plot for the relationship between vitamin D level and bronchopulmonary dysplasia. Due to the small sample size, the publication bias cannot be determined with inspection of the funnel plot.
(TIF)

S4 Fig Sensitivity analysis (4–1) and cumulative analysis (4–2) for the relationship between vitamin D level and bronchopulmonary dysplasia. Std; standardized, BPD; bronchopulmonary dysplasia
(TIF)
