# Supplementary material for: Association between vitamin D level and bronchopulmonary dysplasia: A systematic review and meta-analysis
Source: PLoS One. 2020 Jul 6;15(7):e0235332. doi: 10.1371/journal.pone.0235332 (PMC7337306; doi:10.1371/journal.pone.0235332)
Supplement: S1 Table — (DOCX) [file pone.0235332.s001.docx]

**Supplement** 1. **MEDLINE search strategy**

Database: Pubmed < date of search: June 4^th^, 2019>

Search Strategy:

| **NO.** | **Search Query** | **Results** |
| --- | --- | --- |
| #1 | "Infant, Premature"[Mesh] | 52,939 |
| #2 | "Infant, Low Birth Weight"[Mesh] | 32,673 |
| #3 | (Infant[TW] OR Infants[TW] OR Neonat*[TW]) AND (Prematur*[TW] OR Preterm*[TW]) | 118,409 |
| #4 | "low birth weight"[TW] OR "Extremely Low Birth Weight"[TW] OR newborn*[TW] OR Neonate*[TW] OR "ELBW"[TW] OR "VLBW"[TW] OR "LBW"[TW] | 760,703 |
| **#5** | **#1 OR #2 OR #3 OR #4** | **775,891** |
| #6 | "Vitamin D"[Mesh] | 56,197 |
| #7 | "25-hydroxyvitamin D" [Supplementary Concept] | 6,634 |
| #8 | "Vitamin D"[TW] OR "VitaminD"[TW] OR "VitD"[TW] OR "25-hydroxyvitamin D"[TW] OR "25-hydroxyergocalciferol"[TW] OR Ergocalciferol*[TW] OR Cholecalciferol*[TW] OR Hydroxycholecalciferol*[TW] OR Calcifediol[TW] OR Dihydroxycholecalciferol*[TW] OR "25(OH)D"[TW] OR "1,25(OH)2-vitD"[TW] | 76,045 |
| **#9** | **#6 OR #7 OR #8** | **82,498** |
| #10 | "Bronchopulmonary Dysplasia"[Mesh] | 4,316 |
| #11 | Bronchopulmon*[TW] AND Dysplasia*[TW] | 7,435 |
| #12 | "Lung Diseases"[Mesh] AND "Chronic Disease"[Mesh] | 25,383 |
| #13 | Chronic*[TW] AND (lung[TW] OR pulmon*[TW]) AND (disease[TW] OR diseases[TW] OR injur*[TW]) | 120,252 |
| **#14** | **#10 OR #11 OR #12 OR #13** | **134,800** |
| **#15** | **#5 AND #9 AND #14** | **39** |
