# Supplementary figures and images for: Association between vitamin D level and bronchopulmonary dysplasia: A systematic review and meta-analysis
Source: PLoS One. 2020 Jul 6;15(7):e0235332. doi: 10.1371/journal.pone.0235332 (PMC7337306; doi:10.1371/journal.pone.0235332)

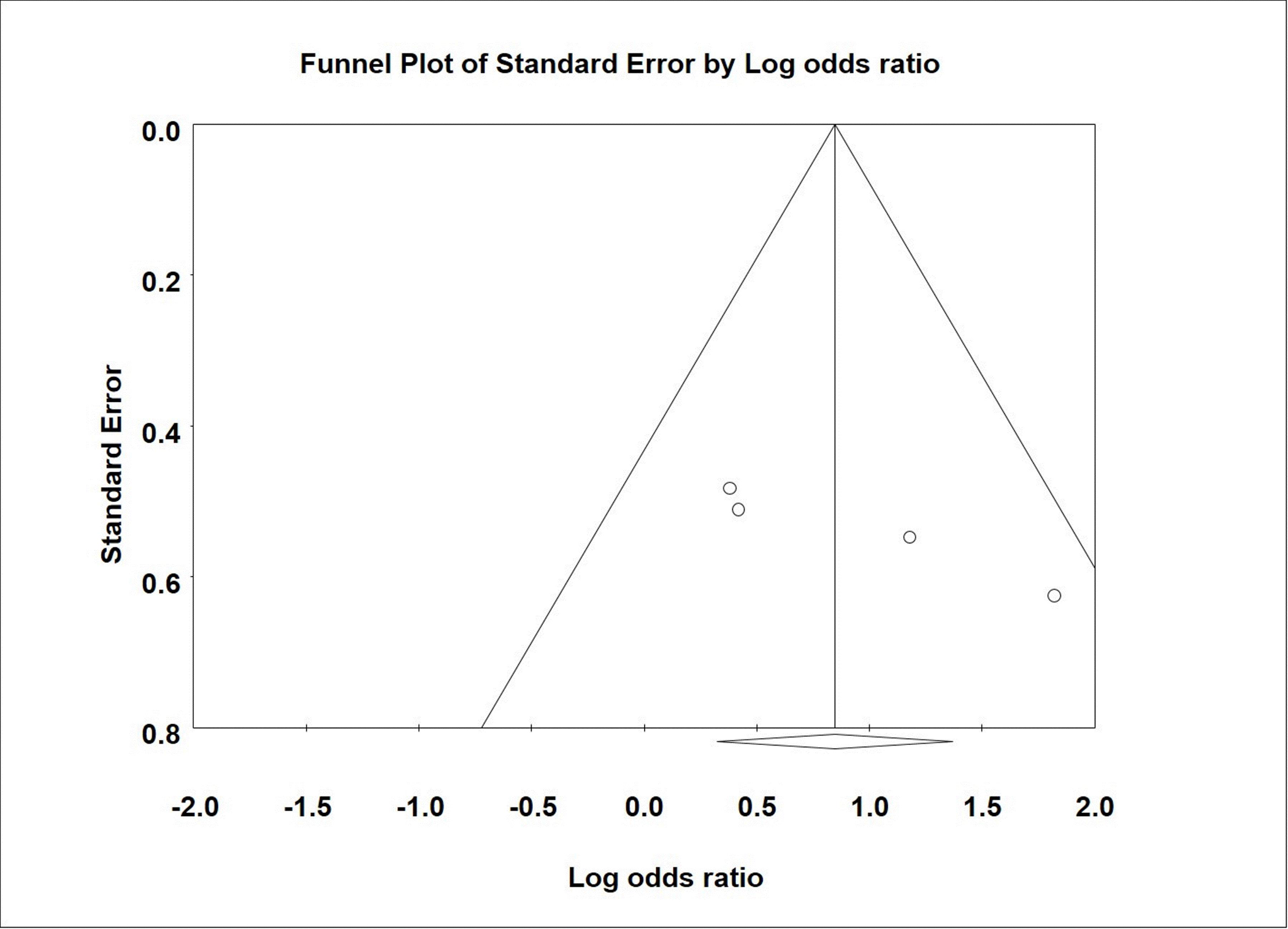

Supplement: S1 Fig — An asymmetrical funnel plot is displayed. (TIF) [file pone.0235332.s002.tif]

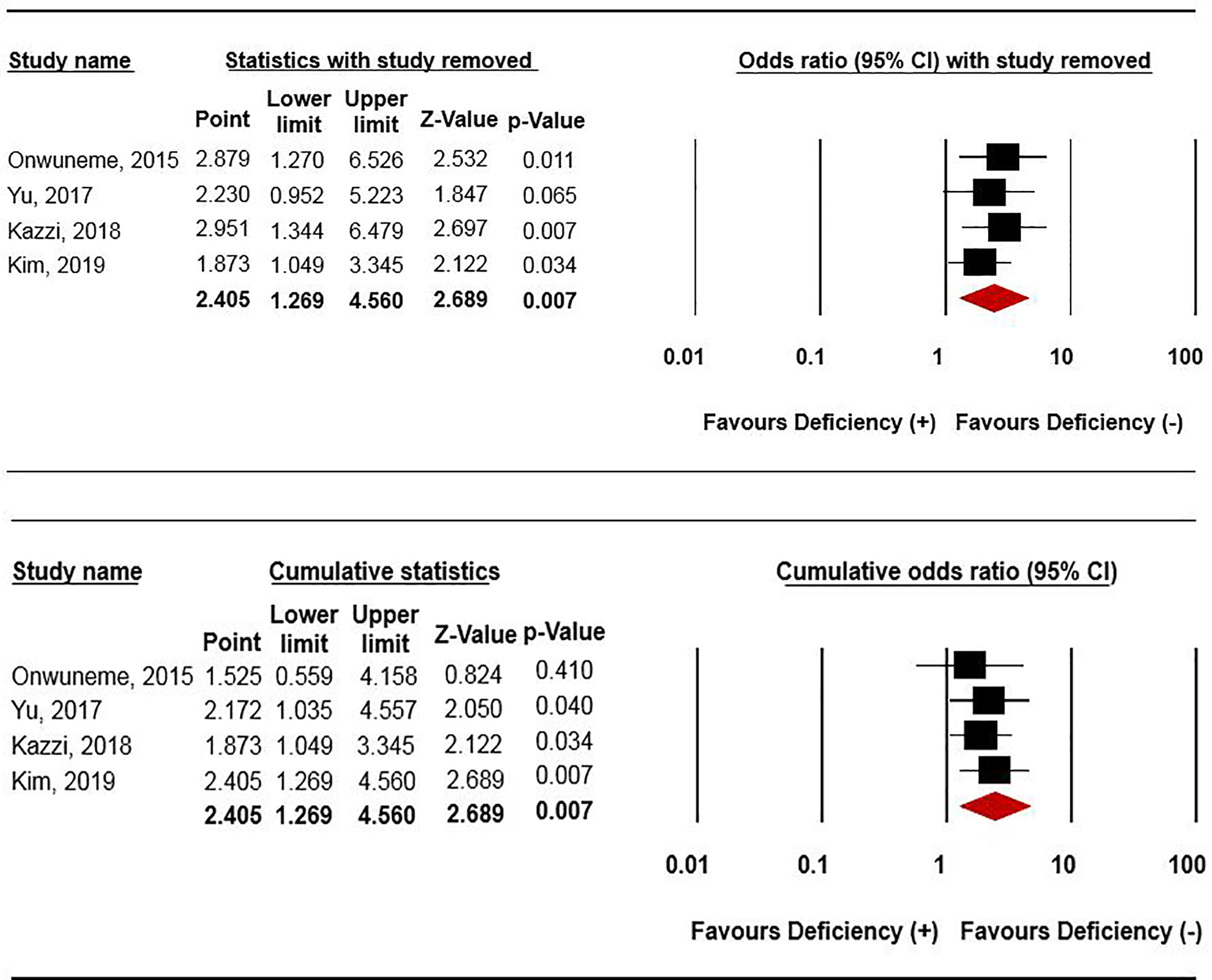

Supplement: S2 Fig — (TIF) [file pone.0235332.s003.tif]

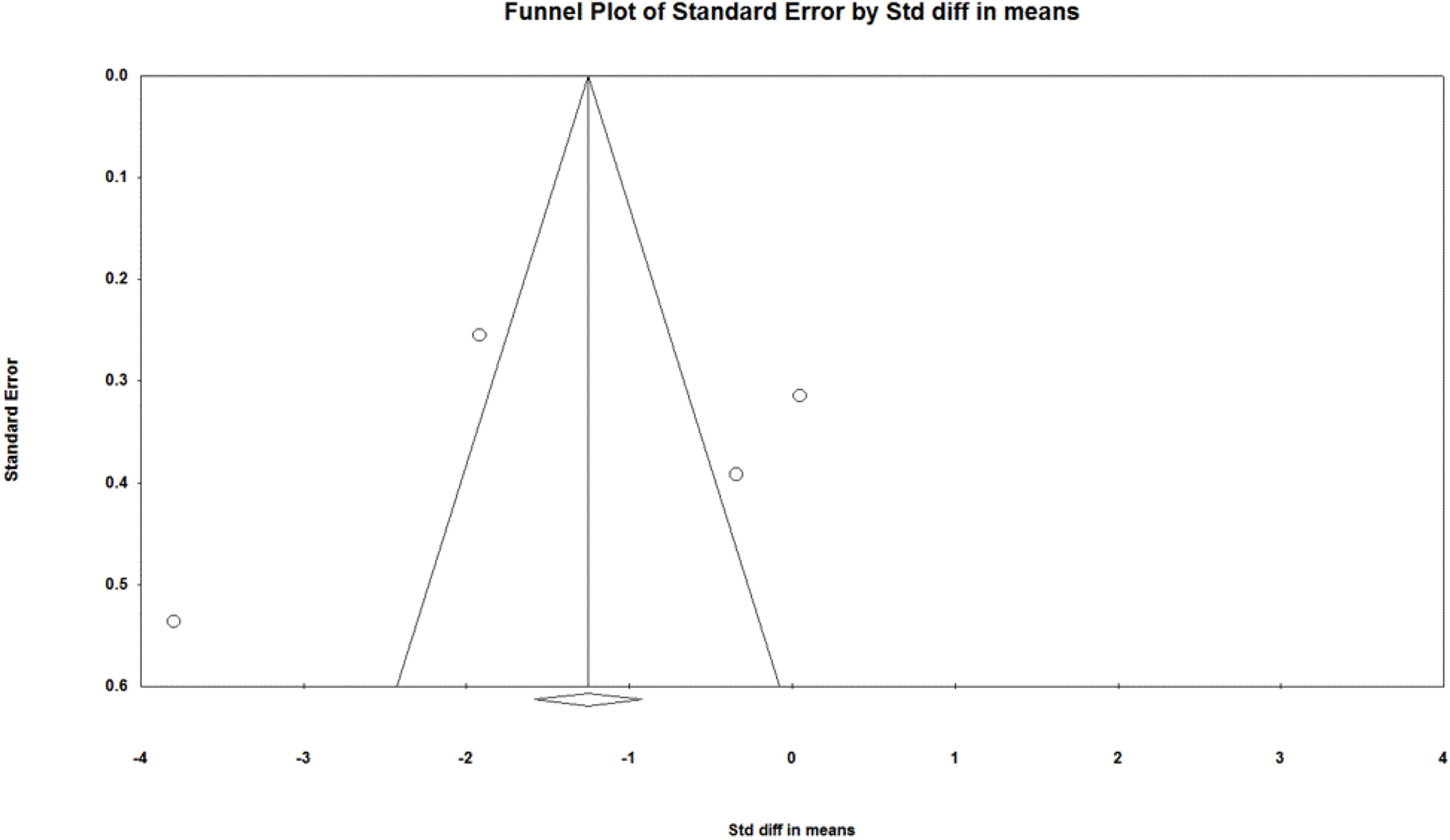

Supplement: S3 Fig — Due to the small sample size, the publication bias cannot be determined with inspection of the funnel plot. (TIF) [file pone.0235332.s004.tif]

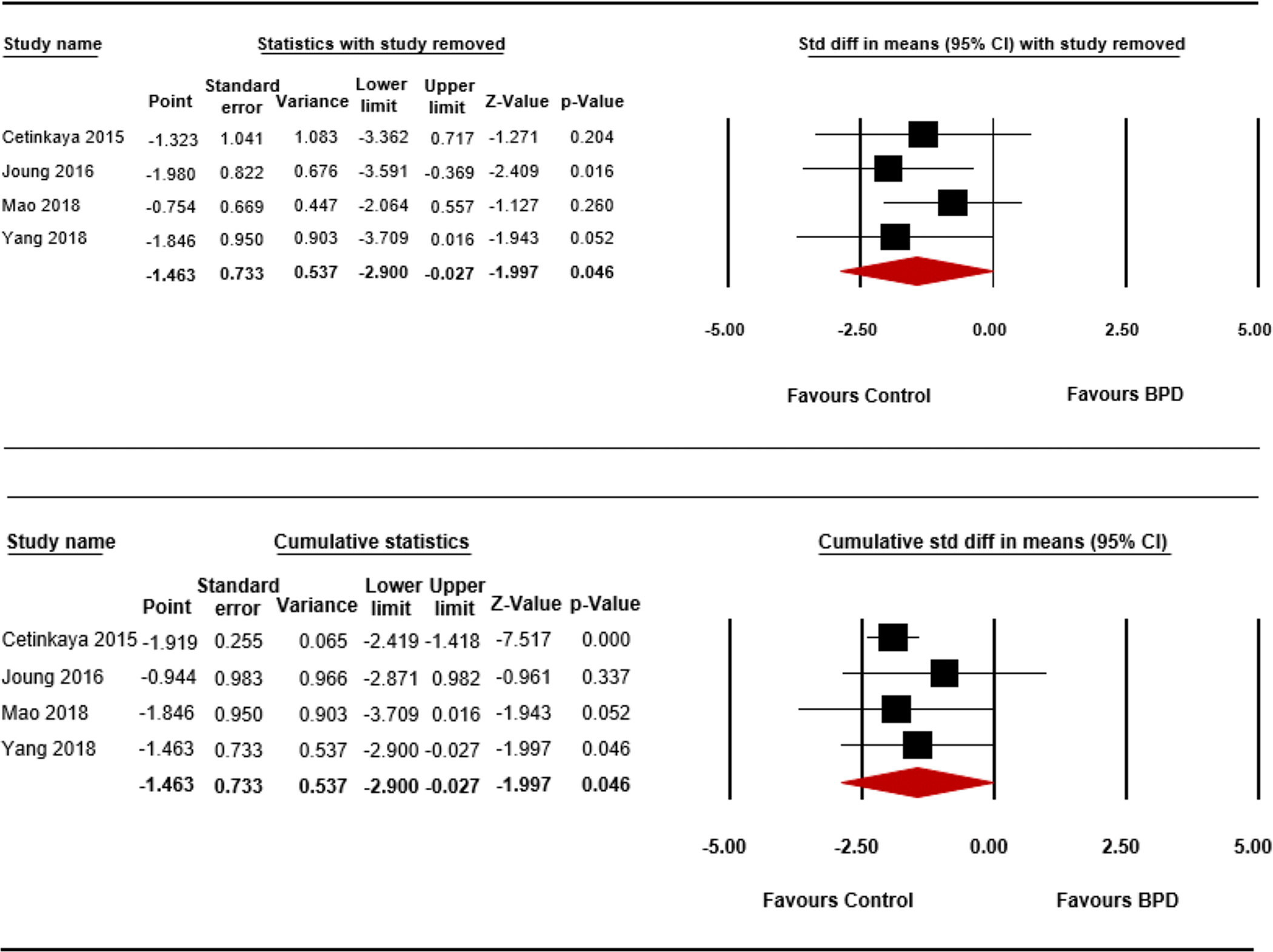

Supplement: S4 Fig — Std; standardized, BPD; bronchopulmonary dysplasia (TIF) [file pone.0235332.s005.tif]
